# MRS4Brain: a software for preclinical proton and deuterium-based MR spectroscopic imaging data

**DOI:** 10.1007/s10334-026-01351-w

**Published:** 2026-04-08

**Authors:** Brayan Alves, Tan Toi Phan, Guillaume Briand, Alessio Siviglia, Gianna Nossa, Jessie Mosso, Eloïse Mougel, Jamie Near, Thi Ngoc Anh Dinh, Omar Zenteno, Bernard Lanz, Thanh Phong Lê, Cristina Cudalbu

**Affiliations:** 1 CIBM Center for Biomedical Imaging, Lausanne, Switzerland; 2 CIBM Pre-Clinical Imaging Section, EPFL - École Polytechnique Fédérale de Lausanne, Lausanne, Switzerland; 3 MRS4Brain Group, School of Basic Sciences, EPFL - École Polytechnique Fédérale de Lausanne, Lausanne, Switzerland; 4 Bernard and Irene Schwartz Center for Biomedical Imaging, Department of Radiology, New York University Grossman School of Medicine, New York, NY USA; 5 Physical Sciences, Sunnybrook Research Institute, Toronto, ON Canada; 6 Department of Medical Biophysics, Temerty Faculty of Medicine, University of Toronto, Toronto, ON Canada

**Keywords:** Preclinical magnetic resonance spectroscopic imaging, Deuterium metabolic imaging, Magnetic resonance spectroscopy, Brain metabolism, Data processing

## Abstract

**Objectives:**

Magnetic resonance spectroscopic imaging (MRSI) is a non-invasive technique for probing metabolism. MRSI enables spatial mapping of metabolite distributions, offering insights into regional metabolic heterogeneity that single-voxel spectroscopy (SVS) cannot capture. However, MRSI produces large multidimensional datasets and requires complex processing pipelines, limiting reproducibility and accessibility. While human studies benefit from advanced processing tools, similar developments in preclinical research remain scarce, highlighting a demand for practical tools accessible to non-experts. Furthermore, recent advances in deuterium-based MRSI have opened metabolic pathway studies, introducing additional dimensions for kinetic information and specific positional labeling, thus substantially increasing dataset complexity and analysis demands.

**Methods:**

To address these needs, we introduce the *MRS4Brain Toolbox*, a freely available MATLAB-based platform for preclinical MRSI supporting proton, deuterium, and other nuclei, with extended functionalities for SVS and diffusion-weighted spectroscopy.

**Results:**

The toolbox integrates reconstruction, preprocessing, quantification, quality control, brain segmentation automatically overlaid on metabolite maps, modeling, and statistical analysis into unified workflows accessible via a graphical interface.

**Conclusion:**

By streamlining data processing and reducing technical barriers, *MRS4Brain Toolbox* promotes reproducibility, harmonization, and broader adoption of basic and advanced spectroscopic techniques in preclinical studies, ultimately facilitating translational metabolic research.

**Supplementary Information:**

The online version contains supplementary material available at https://doi.org/10.1007/s10334-026-01351-w.

## Introduction

Magnetic resonance spectroscopy (MRS) is a non-invasive, versatile, and well-established technique for studying metabolism. It serves as the foundation for magnetic resonance spectroscopic imaging (MRSI) and advanced approaches such as diffusion-weighted spectroscopy (DWS). MRSI builds upon single-voxel spectroscopy (SVS) by enabling spatial mapping of metabolite distributions within a single acquisition, thereby providing insights into regional metabolic heterogeneity. This spatial dimension is particularly important as metabolic alterations often exhibit localized patterns that SVS alone cannot adequately capture. With the ability to simultaneously acquire spectra in different locations, MRSI has become a key player in numerous human studies, such as research on glioma or sclerosis [1–5], and has recently attracted interest in preclinical research [6, 7]. These advancements underscore the increasing recognition of MRSI as a powerful modality for understanding the underlying metabolism in disease progression. Moreover, recent advances in MRSI of deuterium-labeled compounds following metabolism of deuterated tracers have enabled the detailed investigations of metabolic pathways, providing additional kinetic insights and specific positional labeling that further enrich the multidimensional datasets to be dealt with [8–11].

Despite its advantages, MRSI acquisitions inherently produce large, multidimensional datasets, as the number of spectra scales with the phase-encoding steps. This complexity necessitates robust visualization and interpretation strategies to ensure reproducibility and clarity. A major challenge common to all MRS methods is the complexity of the data processing pipeline, which requires extensive knowledge of acquisition and multiple preprocessing steps. These steps often involve different tools to achieve a thorough and effective analysis [12, 13]. As methodological advances continue to push the boundaries of MRS capabilities, the complexity of data processing increases, introducing heterogeneities across studies and making harmonization even more difficult. Without unified workflows, these challenges can hinder reproducibility and slow down the adoption of advanced techniques.

Recent consensus on MRS has provided a framework for standardizing essential steps for spectroscopic data analysis quantification [14–18]. Consequently, substantial efforts have been made in the human research realm, where the emergence of MRS tools, such as Osprey [13], FID-A [19], FSL-MRS [12], Gannet [20], ORYX-MRSI [21], and MRSpa [22], has led to the wider spread of the MRS methodologies. To the best of our knowledge, such advanced developments remain scarce in the preclinical research domain. With the recent advent of free induction decay (FID)-MRSI in preclinical research [6], there is a demand for practical tools that enable harmonized analyses and facilitate dissemination to non-MRSI experts. Bridging this gap is critical for accelerating translational research and aligning preclinical findings with clinical studies.

This paper introduces the *MRS4Brain Toolbox*, a freely available MATLAB-based platform specifically designed for Bruker preclinical spectroscopy studies. It offers automatic workflows via a graphical user interface, aiming to enhance accessibility for researchers starting in the preclinical spectroscopy field. By integrating reconstruction, preprocessing, quality control, quantification, modeling, brain segmentation automatically overlaid on metabolite maps, and statistical analysis into a unified environment, the toolbox streamlines complex processing steps and supports comprehensive analysis of preclinical proton (^1^H) and deuterium (^2^H)-based MRSI datasets. As a secondary aim, the toolbox functionalities were also extended to include SVS and DWS data processing. Ultimately, *MRS4Brain Toolbox* seeks to promote reproducibility, reduce technical barriers, and foster broader adoption of advanced spectroscopic techniques in preclinical research. By making these tools broadly accessible, we aim to facilitate the wider implementation of preclinical MRSI and contribute to more consistent and reproducible experimental outcomes.

## Methods

**Fig. 1:**
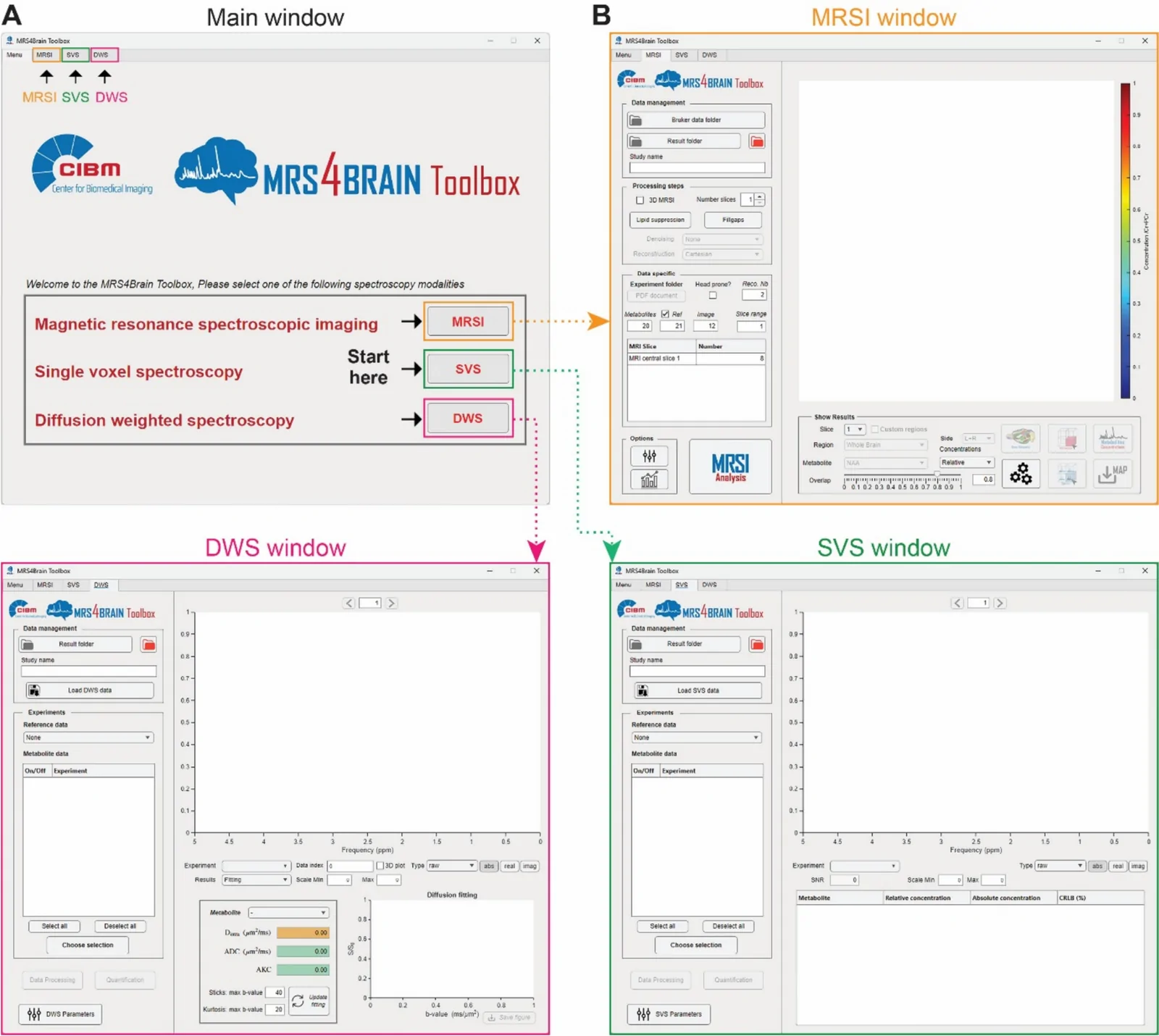
Overview of the *MRS4Brain Toolbox.* **A** Main window of the toolbox. **B** Graphic user interfaces for MRSI (top-right, window highlighted in orange), SVS (bottom-right, window highlighted in green), and DWS (bottom-left, window highlighted in pink)

The current version of the *MRS4Brain Toolbox* is available on the GitHub [23] and Zenodo [24] repositories, providing functionalities for MRSI, SVS, and DWS (Fig. 1). Detailed methods are described in the following sections and Supplementary Table 1, while the installation guidelines are provided in the Supplementary Information.

### MRSI processing pipeline

#### Processing methods

**Fig. 2:**
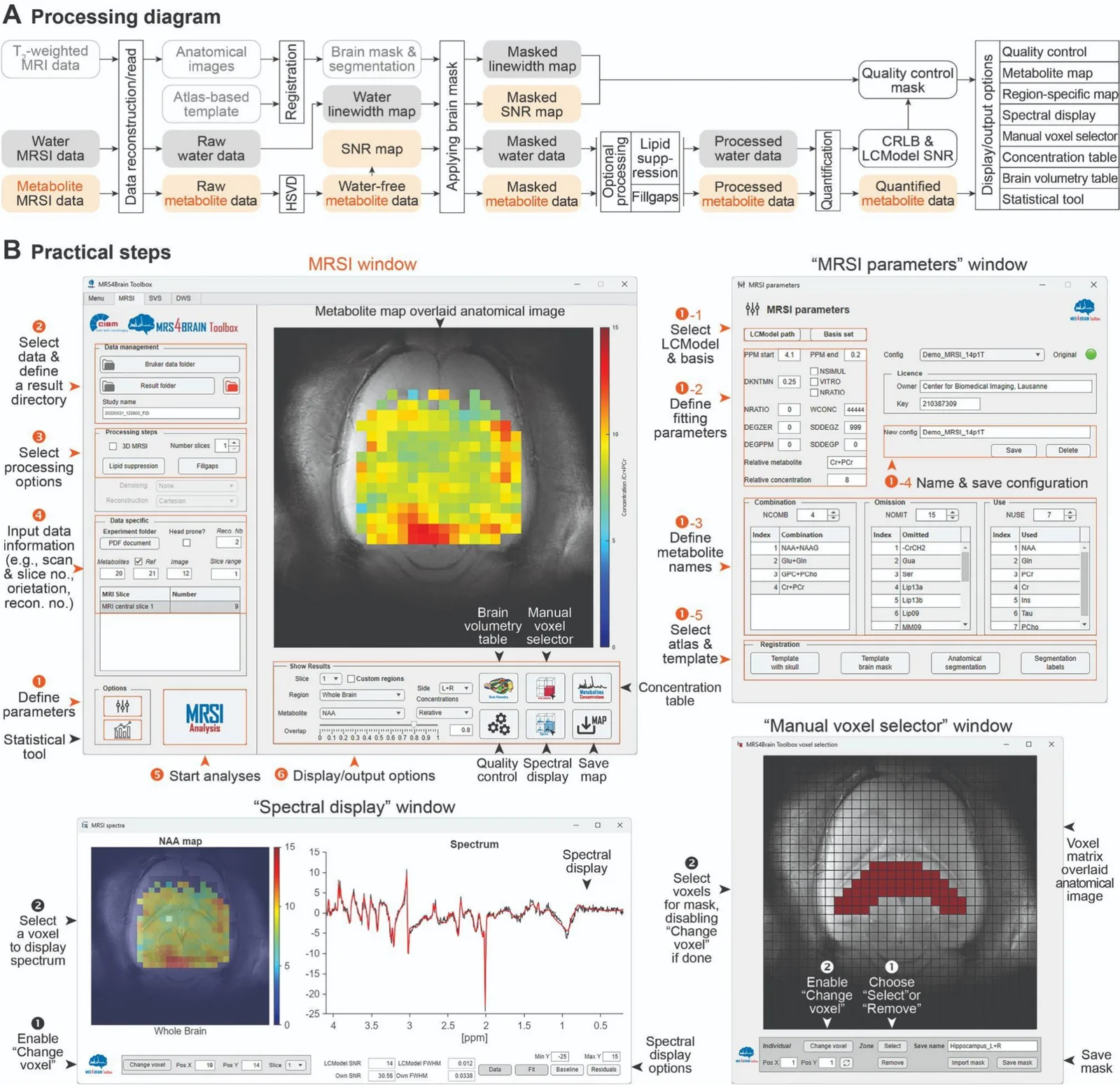
Processing pipeline and practical steps for MRSI data. **A** Processing pipeline diagram from data reconstruction, processing to result display, and outputs. **B** Typical workflow for processing an MRSI dataset. Top-left: main MRSI window displays steps 1–6, guiding users through parameter configuration, data input, processing option selection, and display/output choices. Top-right: *MRSI Parameters* window (step 1) for defining parameter configurations. Bottom-left: *Spectral display* window for visualizing spectra in individual voxels. Bottom-right: *Manual voxel selector* for defining user-specified regions of interest

The MRSI processing pipeline (Fig. 2A) takes magnetic resonance imaging (MRI) images (here, T_2_-weighted coronal images), metabolite, and water MRSI data as inputs. The toolbox can effectively read data reconstructed and provided by various versions of Bruker ParaVision 360 software (versions 1.x to 2.0, and 3.0 and above). MRI data need to be in *NIfTI* format (*.nii or *.nii.gz), while MRSI data can be read as a *.fid file (versions v1.x to v2.0) or a *.fid_proc.64 (versions v3.0 and above). Data selection can be managed in the *Data management* and *Data specific* panels of the MRSI window.

##### Co-registration

One key motivation for combining anatomical information from MRI data is the ability to segment the different brain regions and generate precise anatomical masks. These masks can then be applied to the MRSI dataset to examine the metabolite contents within specific brain regions. To achieve this, a co-registration between a template with atlas-based segmentation and the acquired MRI images is done using Advanced Normalization Tools (ANTs) [25]. This process employs rigid, affine, and symmetric normalization transformation methods in ANTs to align the template with the MRI image, yielding a transformation matrix that is used to accordingly transfer the brain mask and segmentation to the MRI image space. The toolbox package includes two home-made rat brain templates for 9.4 T and 14.1 T field strengths. These templates were generated from T_2_-weighted Turbo-Rapid acquisition with relaxation enhancement (Turbo-RARE) images (see the Demonstration data acquisition section for details) and incorporate segmentations based on the SIGMA rat brain atlas [26]. Users also have the flexibility to use their own templates and segmentation schemes if desired.

##### Preprocessing

The MRSI data preprocessing starts right after the MRI registration steps, and both are completely decorrelated (Fig. 2A). The current version of the toolbox can handle different types of data available in the Bruker system, such as FID-MRSI, spin echo (SE)-MRSI, and point-resolved spectroscopy (PRESS)-MRSI, as long as they are reconstructed via the ParaVision software. First, metabolite MRSI data undergo water signal removal using the Hankel singular value decomposition (HSVD) method [27]. The resulting water-free metabolite data are then used for further processing steps, including the computation of a signal-to-noise ratio (SNR) map using the metabolite N-acetylaspartate (NAA). In particular, the NAA signals within 1.9 to 2.1 ppm are used, with the noise estimated from the signals within -0.5 to 0.5 ppm. Subsequently, water MRSI data are processed to generate a linewidth map and a water power map. These SNR and linewidth maps serve as key metrics for quality control later in the processing pipeline. To exclude non-brain regions, both metabolite and water data are masked using the brain mask obtained from the co-registration step. Two optional preprocessing steps are provided in the *Processing steps* panel: one is retrospective *lipid suppression*, implemented based on singular value decomposition (SVD). This approach assumes that lipid and metabolite signals are orthogonal in the time or frequency domain and do not spatially overlap [28, 29]. The process begins by delineating brain and scalp regions using a mask of the water power map. SVD is then applied to the scalp voxels to construct an orthogonal basis representing lipid components. The rank of this basis is determined by assessing the energy ratio between brain and scalp regions after applying the corresponding projection operator as E_brain_/E_Skull_ ≥ α (default: α = 0.8). It is noted that in the toolbox, *lipid suppression* only applies to metabolite data. Another method is backward linear prediction (called *Fillgaps* in the toolbox), implemented with an autoregressive algorithm (MATLAB’s *fillgaps* function) to reconstruct data points missing due to the inherent acquisition delay (AD) in FID-MRSI sequences. The number of missing points in FID is determined by the acquisition bandwidth (BW) and the AD time, and the prediction is applied to the FID signals [30]. *Fillgaps* applies to both metabolite and water data to ensure they are the same length in the quantification process. Once the processing is successfully performed, each spectrum corresponding to each voxel location in both metabolite and water data is saved into a *.RAW file for LCModel fitting.

##### Fitting and quantification

Fitting and quantification use the LCModel software [31] with a basis set containing sequence-specific simulated spectral profiles of metabolites and in vivo-measured macromolecules, specific to each acquisition sequence [32] (see details for basis sets in the Demonstration data acquisition section and Supplementary Table 2). We are using in vivo-measured macromolecules, specific to each acquisition sequence, as described in previous studies [6, 32–35]. The fitting process starts with the generation of *.CONTROL files containing data specification and all LCModel parameter configurations, some of which are specified in the *MRSI parameters* window (see the Practical guidelines section of the MRSI processing pipeline for details). Then LCModel uses all information in the *.CONTROL file to handle the fitting and quantification. Upon successful processing, output files such as *.coord, *.ps, and *.table are generated and stored in the results directory. It is necessary to note that although the methods presented in this work are primarily described for ^1^H-MRSI data without and with ^2^H-glucose (Glc) infusion (i.e., indirect ^2^H-FID-MRSI data), the toolbox is capable of handling X-nuclei MRSI data. Although the implementation for X-nuclei acquisitions should be straightforward, the processing pipeline must be carefully adapted for each specific nucleus (e.g., usage of water and lipid suppression, metabolite basis sets, LCModel control files, etc.), followed by thorough testing and validation.

#### Practical guidelines

To start using the MRSI functionalities, users can click the *MRSI* button in the main window (Fig. 1). Alternatively, selecting the MRSI tab in the top‑left corner (Fig. 1) also opens the MRSI window. Using FID and SE demonstration datasets acquired at 9.4 T and 14.1 T, detailed in the Demonstration data acquisition section, the typical processing workflow is illustrated in Fig. 2B (top-left) and comprises the following steps:

*Step 1*: Open the *MRSI Parameters* window by selecting the *MRSI Parameters* button (Fig. 2B, top-right).

1. Specify the directory containing the installed LCModel and select the appropriate metabolite basis set for the data type. For *Fillgaps* in particular, the basis set assuming no first-order phase effects (i.e., no AD) is needed to fit spectra reconstructed by the backward linear prediction. The metabolite basis sets are provided with demonstration datasets (Supplementary Table 2).
2. Define LCModel parameters such as controlling baseline flexibility (DKNTMN, default: 0.25), prior phasing information such as DEGZER (default: 0), SDDEGZ (default: 999), DEGPPM (default: 0), SDDEGP (default: 0), and fitting ppm range specified in the configuration file (default: starting from 4.1 to 0.2 ppm), NRATIO (default: 0), WCONC (default: 44444), the internal reference metabolite (default: Cr+PCr), and its concentration (default: 8). Default parameters defined in the toolbox were used for ^1^H-MRSI data, except for SDDEGP = 5 for 9.4 T and SDDEGP = 0 for 14.1 T datasets. In analyzing the indirect ^2^H-FID-MRSI data at 9.4 T, DKNTMN = 0.15, SDDEGP = 5, and fitting ppm range from 4.1 to 1.8 ppm were set, while all other parameters were left at their default values in the toolbox. It is noted that DKNTMN, DEGZER, DEGPPM, SDDEGZ, SDDEGP, NRATIO, and WCONC are all parameters used by the LCModel algorithm in the control files to fit the data. Details of each parameter can be found in the LCModel User’s Manual (https://s-provencher.com/lcm-manual.shtml).
3. Configure metabolite handling within the basis set (e.g., usage, combination, or omission).
4. Save the configuration to a file. Note that using an existing filename will overwrite the previous configuration; assigning a new name prevents overwriting.
5. Select template and atlas for anatomical co-registration. Template, brain mask, anatomical segmentation, and their labels for each field strength (9.4 T and 14.1 T) are included in the toolbox package.

*Step 2*: Specify the directories for data input and result output. It is important to assign unique study names (i.e., result folder names) for different analyses to prevent overwriting.

*Step 3*: Select processing options. There are two preprocessing options for two-dimensional (2D) or three-dimensional (3D) data: *lipid suppression* and backward linear prediction (*Fillgaps*). If data are acquired from a 3D MRSI sequence, the *3D MRSI* checkbox and the number of slices should be specified.

*Step 4*: Input the data information, such as MRI, metabolite, and reference water MRSI scan numbers, slice number for brain regional anatomy, reconstruction number, the number of slices averaged for the display, and brain image orientations when default magnet orientations differ across scanners (e.g., *Head prone* or *tail prone* in the Bruker system). For demonstration data in this work, we used the center slice of the MRSI slab for brain regional anatomy, reconstruction number 2, which applies Hamming *k*-space filtering, and only selected the *Head prone* checkbox for 9.4 T data.

*Step 5*: Run the analysis. Several message dialog boxes appear to inform the progress: co-registration, HSVD, linewidth, SNR, preprocessing steps such as *lipid suppression* and *Fillgaps* if specified, and saving data to *.RAW files for LCModel, quantification using LCModel, creation of a metabolite map, etc. After completing the analysis, the relative concentration NAA map for the whole-brain slice is the default display overlaid on the anatomical image.

*Step 6*: Explore display and output options (Fig. 2B, top-left). The *Quality control* is to control the voxels that appear in the map using metabolite SNR (from NAA-based estimation or LCModel output), reference water linewidth, and Cramer-Rao lower bound (CRLB) percentage [17] (see descriptions for data quality metrics in Supplementary Information). The color map scaling is also set in this function. For demonstration purposes, we applied SNR ≥ 10, linewidth ≤ 1.25 times the averaged linewidth, and CRLB < 30%. A specific metabolite map for a specific brain region or whole-brain slice can be optionally chosen by selecting one from the *Metabolite* and *Region* dropdown menus. Brain mask, water power, SNR, linewidth, zero-order phase, and first-order phase maps are also listed in the *Metabolite* dropdown menu. Additionally, the CRLB map is also possible to display for quality assessment, as it is listed in the *Concentration* dropdown menu. The *Spectral display* window allows the user to inspect spectra at a chosen voxel. To do this, *Change voxel* must be enabled before selecting the desired voxel location (Fig. 2B, bottom-left). This window also provides options to display spectra from spectroscopic *data,* LCModel’s *Fit*, *Baseline,* and *Residuals*; the different quality metrics, such as SNR, linewidth calculated during the processing and by LCModel; and manual axis scaling if needed. The *Manual voxel selector* window is available for defining custom regions of interest, instead of using atlas-based regions for MRSI data masking (Fig. 2B, bottom-left). Depending on the type of LCModel concentration output (“*absolute”* or *relative*) specified in the *Concentration* dropdown menu, the *Concentration table* lists metabolite names, mean concentrations, standard deviations, and voxel counts for the chosen brain region that passed the quality control thresholds. In this study, “absolute” concentration refers to the LCModel concentration estimate in reference to the unsuppressed water signal, with no T_1_ or T_2_ relaxation corrections applied, whereas relative concentration refers to total creatine (tCr) concentration. The *Brain volumetry table* provides calculations of brain areas based on co-registered brain segmentation. Both concentration and brain volumetry tables can be saved as MATLAB data files in the result folder. The *Save map* allows users to store the currently displayed maps in the result folder. Additionally, if toolbox outputs, such as concentration tables, are saved in the result folder, it is possible to perform statistical tests using the *Statistical tool* (Fig. 2B, top-left, and Figure S1). Options to select testing results, metabolite names, type of statistics, and their parameters [e.g., Student’s *t *test, one/two-way analysis of variance (ANOVA), Bonferroni’s multiple comparisons, one/two-sided *p* value], and to display the testing results (i.e., bar graph, result table) are all available (see list of statistical tests in Supplementary Information).

To only view the results of the already processed/quantified data without reprocessing, first, all information, especially data and result directories, study name, data specifications, processing options, and parameter configuration, should be specified the same as when they were previously processed, and then select the *MRSI analysis* button. Two or three message dialog boxes may appear, depending on the status of the toolbox. The message dialog box 1 asks whether to redo image co-registration or to keep the preexisting one. The message dialog box 2 asks whether or not to restart the processing pipeline. The message dialog box 3 asks whether or not to reprocess the data. If the tool is newly launched (i.e., blank in the metabolite map window), message dialog boxes 1 and 3 appear sequentially, and *Keep preexisting* and *No* should be, respectively, chosen. If the tool is in use (i.e., image or map shown in metabolite map window), message dialog boxes 1, 2, and 3 appear sequentially, and *Keep preexisting*, *Yes*, and *No* should be, respectively, chosen. The whole-slice NAA maps relative to tCr will appear as the default if the reloading process is done successfully. 

### SVS processing pipeline

#### Processing methods

**Fig. 3:**
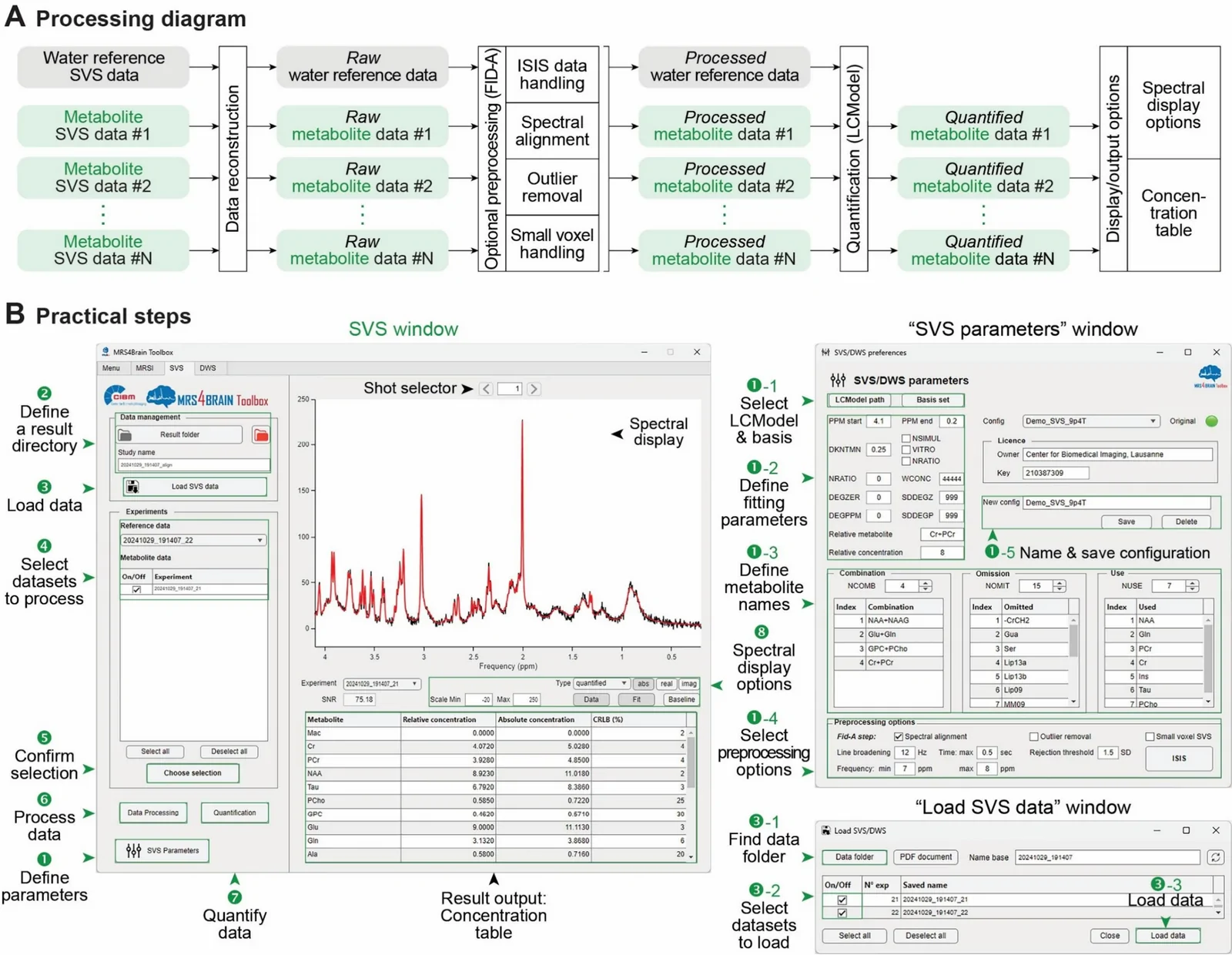
Processing pipeline and practical steps for SVS data. **A** Processing pipeline diagram from data reconstruction, processing to result display and outputs, **B** Typical workflow for processing an SVS dataset. Left: The main SVS window displays steps 1 to 8, guiding users through parameter configuration, data loading and processing, and selecting display and output options. Top-right: The *SVS/DWS Parameters* window (step 1) for defining parameter configurations. Bottom-right: The *Load SVS data* window (step 3), illustrating the procedure for loading datasets

Vendor-reconstructed data (e.g., *fid/ser* files generated by Bruker ParaVision-360) are imported into the toolbox through the *Load SVS/DWS* interface (Fig. 3A). If necessary, the data are automatically reformatted to ensure compatibility with subsequent processing steps. When multiple datasets are provided, each processing step is applied sequentially to every dataset before advancing to the next stage of the workflow. Raw data are then processed using the data processing module, which is adapted from the FID-A framework [19]. This module provides four optional preprocessing features: image-selected in vivo spectroscopy (ISIS) handling, spectral alignment, outlier removal, and small voxel handling. Each feature, along with its associated parameters, can be configured and saved via the SVS/DWS *Parameter* window.

- ISIS handling: For datasets acquired using ISIS localization, the raw data are first separated into two subsets corresponding to odd and even shots within the ISIS scheme. Subsequent processing steps are applied independently to each subset.
- Spectral alignment: All acquisitions are aligned to their median spectrum across the shots (or averages or repetitions) using the NAA peak at 2.01 ppm as a reference. Adjustable parameters include line broadening (default: 12 Hz), frequency range limits (a larger frequency range can be selected if needed, not limited only to the NAA singlet), and maximum alignment time (default: 0.5 s).
- Outlier removal: When data exhibit excessive noise, this feature identifies and excludes outlier shots based on a rejection threshold (default: 1.5 SD) specified in the configuration file. This step can improve overall spectral quality before data combination.
- Small voxel handling: For ISIS data acquired from small voxels with low SNR, this function merges odd and even shots into a single dataset before further processing. Note that this reduces the total number of shots by half. For a reliable alignment of the ON and OFF shots in ISIS, we recommend using this option for all datasets.

Following these steps, the preprocessed data are combined for quantification. LCModel fitting is then performed, with adjustable parameters for controlling baseline flexibility (DKNTMN, default: 0.25), prior phasing information such as DEGZER (default: 0), SDDEGZ (default: 999), DEGPPM (default: 0), SDDEGP (default: 999), and fitting ppm range specified in the configuration file (default: starting from 4.1 to 0.2 ppm), NRATIO (default: 0), WCONC (default: 44,444), the internal reference metabolite (default: Cr + PCr), and its concentration (default: 8). The *.RAW file is prepared as input for LCModel. Upon successful quantification, output files such as *.CONTROL, *.coord, *.ps, and *.table are generated in the results directory. The main SVS window subsequently displays individual fitted spectra and metabolite concentration values in the table. It is noted that fitting and quantification are performed by LCModel; therefore, “absolute” concentration refers to LCModel‑derived concentration estimates referenced to the unsuppressed water signal, with no T_1_ or T_2_ relaxation corrections applied (of note T_1_ and T_2_ relaxation corrections are not applied as we are performing measurements at ultra-short TEs and long TRs), whereas relative concentration refers to values expressed relative to tCr.

#### Practical guidelines

To start using the SVS functionalities, users can click the *SVS* button in the main window (Fig. 1). Alternatively, selecting the SVS tab in the top‑left corner (Fig. 1) also opens the SVS window. Using stimulated echo acquisition mode (STEAM) and spin echo full intensity acquired localized (SPECIAL) [36] SVS demonstration datasets acquired at 9.4 T and 14.1 T, detailed in the Demonstration data acquisition section, the typical processing steps are the following (Fig. 3B, top-left):

*Step 1*: Open the *SVS/DWS Parameters* window by selecting the *SVS Parameters* button (Fig. 3B, top-right).

1. Specify the directory containing the installed LCModel and select the appropriate metabolite basis set for the data type. The metabolite basis sets are provided with demonstration datasets (Supplementary Table 2).
2. Define LCModel parameters (e.g., DKNTMN, DEGZER, SDDEGZ, DEGPPM, SDDEGP, fitting frequency range, etc.), the internal reference metabolite, and its concentration. Default parameters defined in the toolbox were used in this work (see Processing methods section of SVS processing pipeline for default values).
3. Configure metabolite handling within the basis set (e.g., usage, combination, or omission).
4. Select preprocessing options. For the STEAM data, we applied spectral alignment with outlier removal. Small voxel and ISIS options are not selected. For the SPECIAL data, we applied ISIS handling, small voxel handling, spectral alignment, and outlier removal. All other parameters remain at default values.
5. Save the configuration to a file. Note that using an existing filename will overwrite the previous configuration; assigning a new name prevents overwriting.

*Step 2*: Specify the directory for output. It is important to assign unique study names (i.e., result folder names) for different analyses to prevent overwriting.

*Step 3*: Open the *Load SVS/DWS data* window by selecting the *Load SVS data* button (Fig. 3B, bottom-right).

- (1) Specify the data directory.
- (2) Select all datasets used for analysis. Options to open an experiment note PDF file and rename data prefixes are available if necessary.
- (3) Complete the process by selecting the *Load data* button.

*Step 4*: Select metabolite datasets for analysis and a water dataset for reference.

*Step 5*: Confirm selection by selecting the *Choose selection* button. If successfully processed, the raw spectrum (Type: *raw*) will appear in the *Spectral display* window. It is possible to inspect the individual shot spectra using the shot selector at the top of the *Spectral display* window.

*Step 6*: Perform preprocessing steps by selecting the *Data processing* button. If successfully processed, a combined spectrum (Type: *processed* + *sum*) will appear in the *Spectral display* window. It is possible to review the processed data before combination by selecting *processed* in the *Type* dropdown menu and using the shot selector.

*Step 7*: Perform quantification by selecting the *Quantification* button. If successfully quantified, fitted spectra (Type: *quantified*) will display alongside data in the *Spectral display* window.

*Step 8*: Options to visualize and output results. Spectra could be displayed in absolute (*abs*), real (*real*), or imaginary (*imag*) formats for raw, processed, and combined data. For quantified data, additional options such as *Data*, *Fit*, and *Baseline* are available. Results, including spectra and concentration tables from different datasets, could be viewed by selecting the corresponding dataset name in the *Experiment* dropdown menu. All data are stored in the folder named in step 2, which contains the subfolders *raw, processed,* and *quantified,* corresponding to steps in the pipeline. If *Outlier removal* is used, text files containing removed shot numbers are also saved in the folder.

To only view the results of the already processed/quantified data without reprocessing, first, it is necessary to specify the data and result directories. Once the study name is entered correctly, the toolbox will automatically reload the existing results. When the fitted spectrum is overlaid with the SVS data in the *Spectral display* window, this confirms that the results have been successfully reloaded.

### DWS processing pipeline

#### Processing methods

**Fig. 4:**
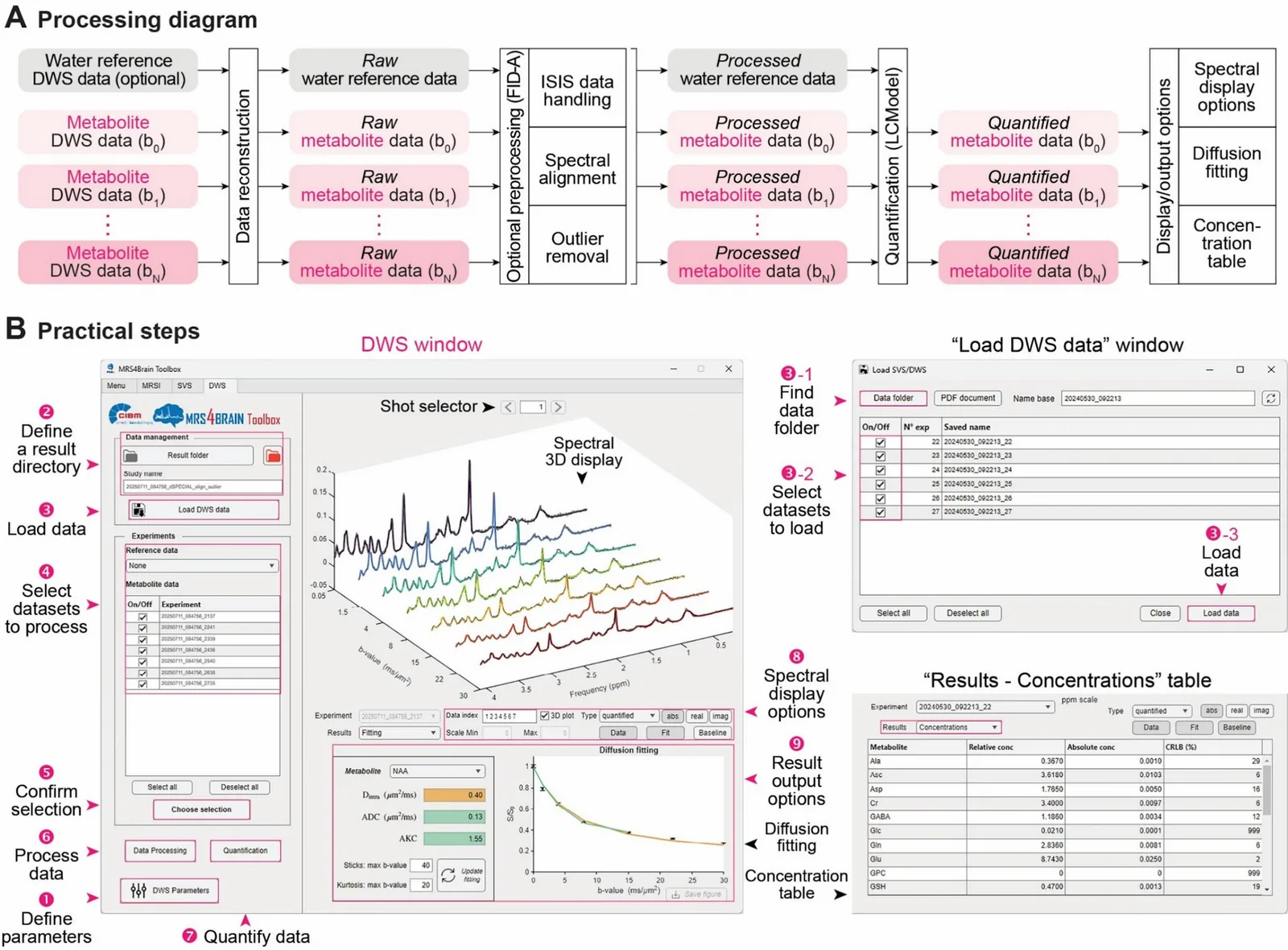
Processing pipeline and practical steps for DWS data. **A** Processing pipeline diagram from data reconstruction, processing to result display, and outputs, **B** Typical workflow for processing a DWS dataset. Left: The main DWS window shows steps 1 to 9, guiding users through parameter configuration, data loading and processing, and selecting display and output options. Top-right: The *Load DWS data* window (step 3) demonstrates the procedure for loading datasets. Bottom-right: The “concentration” table is an output option, alongside diffusion fitting

The DWS data processing pipeline (Fig. 4A) is adapted from the work described by Mosso et al. [37] and closely follows the structure of the SVS framework. It employs the same *Load SVS/DWS* interface for data import and the *SVS/DWS Parameters* window for defining processing parameters. Preprocessing capabilities include ISIS handling, spectral alignment, and outlier removal, followed by quantification using LCModel. For spectral visualization, in addition to displaying individual spectra, the pipeline supports 3D plotting of all DWS-fitted spectra across different b-values, enabling assessment of signal attenuation as a function of b-value for a given diffusion time. Similar to the SVS workflow, a “concentration” table is provided as one of the output options. If the reference dataset, such as water data, is not specified, this table contains values in arbitrary units, not concentrations. An additional feature is diffusion fitting, which estimates diffusion properties for each metabolite. This functionality is implemented using randomly oriented stick [38] and kurtosis [39] models. It should be noted that the b-values displayed in the toolbox are extracted directly from the scan parameters specified in the method file. Corrections for factors such as cross-term interactions must be estimated externally to obtain accurate final results [40].

#### Practical guidelines

To start using the DWS functionalities, users can click the *DWS* button in the main window (Fig. 1). Alternatively, selecting the DWS tab in the top‑left corner (Fig. 1) also opens the DWS window. Using diffusion-weighted SPECIAL (dSPECIAL) [37] DWS demonstration datasets acquired at 9.4 T and 14.1 T, detailed in the Demonstration data acquisition section, the typical processing steps are the following (Fig. 4B, top-left):

*Steps 1 to 7* are closely aligned with steps 1–7 described in the practical guidelines for SVS data (Fig. 4B, top-left and top-right). For preprocessing options in step 1, we selected ISIS handling, spectral alignment, and outlier removal. All other parameters remain at default values.

*Step 8*: Options to visualize results. In addition to step 8 described in the practical guidelines for SVS data, 3D plotting is available in the DWS processing pipeline to visualize signal attenuation across b-values. When the *3D plot* option is selected, all spectra are displayed in 3D, with options to show *Data*, *Fit*, and *Baseline* also supported in this view. Datasets are automatically sorted in ascending order by name; however, if data acquisitions at different b-values were performed in random order, they can be manually rearranged in ascending b-value order using the *Data Index* box for display. Unlike SVS, DWS spectra, particularly at high b-values, exhibit a highly reduced SNR due to the influence of diffusion gradients or motions. Therefore, it is essential to evaluate individual shots at each processing step using the shot selector.

*Step 9*: Options to output the results. Similar to the SVS processing pipeline, the “concentration” table displays both LCModel relative and “absolute” concentrations, along with CRLB values for each metabolite (Fig. 4B, bottom-right). The diffusion fitting feature offers additional tools to examine diffusion properties of each metabolite in the *Metabolite* dropdown menu, either through intra-stick diffusivity (D_intra_), apparent diffusion coefficient (ADC), and apparent kurtosis coefficients (AKC), or by viewing fitted curves. It is possible to restrict the maximum b-values for diffusion fitting models using the corresponding input fields and to apply changes by clicking the *Update fitting* button. To assist this, the CRLB values in the table can help determine an appropriate range of b-values or identify which metabolites are accurately fitted.

### Demonstration data acquisition

Wistar adult rats (Charles River Laboratories, L’Arbresle, France) under 1.5 to 2.5% isoflurane anesthesia were used for in vivo acquisitions. The protocol was approved by the Committee on Animal Experimentation in the Canton of Vaud, Switzerland. The body temperature of the animals was kept at 37.5 ± 1.0 °C by circulating warm water and measured with a rectal thermosensor. The respiration rate and body temperature were monitored using a small-animal monitor system (SA Instruments, New York, USA). During data acquisitions for MRI, MRSI, SVS, and DWS, all animals were placed in an in-house-built or a Bruker-manufactured holder, with their heads fixed in a stereotaxic system using a bite bar and a pair of ear bars.

Each type of demonstration ^1^H data was acquired at two field strengths (9.4 T and 14.1 T), while indirect ^2^H-FID-MRSI data were acquired at 9.4 T. The horizontal 9.4 T magnet (Magnex Scientific, Yarnton, UK) has a 660 mT/m peak strength and 4570 T/m/s slew rate shielded gradient set (Bruker B-GA12S HP) interfaced to a Bruker console (BioSpec AVANCE NEO, ParaVision 360 v3.5), and MR data were acquired using a quadrature volume transmit coil and a four-channel cryogenic receive coil specialized for rat head (CryoProbe, Bruker). The horizontal 14.1 T magnet (Magnex Scientific, Yarnton, UK) has a 1 T/m peak strength and 5500 T/m/s slew rate shielded gradient set (Resonance Research, Billerica, USA) interfaced to a Bruker console (BioSpec AVANCE NEO, ParaVision 360 v3.3), and MR data were acquired using a home-made transceiver quadrature surface coil (two 1.8 × 1.6 cm loops, covering a curved 1.8 × 2.7 cm^2^ surface with 14 mm radius of curvature).

For reference, coronal and axial MRI images were acquired on both systems using T_2_-weighted Turbo-RARE image. Scan parameters were repetition time (TR) = 4100 ms, echo time (TE) = 27 ms, number of averages = 10, RARE factor = 6, 128 × 128 matrix, field of view (FOV) = 24 × 24 mm^2^, slice thickness = 0.3 mm (9.4 T) or 0.2 mm (14.1 T). The shimming procedure described by Simicic and Alves et al. [6] and demonstrated by Cudalbu and Alves [41, 42] was used.

For the indirect ^2^H measurement, the preclinical glucose infusion procedure presented by Siviglia et al. [43] was used. A bolus of 99%-enriched [6,6’-^2^H_2_] Glc (Sigma-Aldrich; 20% m/v in saline solution) was administered intravenously over 5 min, via an infusion pump and femoral vein cannulation, and was followed by a continuous infusion of 70%-enriched [6,6’-^2^H_2_] Glc (Sigma-Aldrich; 20% m/v in saline solution) for 2 h. ^1^H-FID-MRSI data were acquired before the ^2^H-Glc infusion (water and metabolite signals) and along the 2-h infusion protocol (metabolite signal only, 9 iterations), to characterize the glutamate + glutamine (Glx) ^2^H-labeling turnover in the rat brain [31].

#### MRSI data

##### FID-MRSI

Metabolite data were acquired from a single slice, mainly covering the hippocampus and striatum. FID-MRSI was used at 9.4 T and 14.1 T for ^1^H and at 9.4 T for indirect ^2^H data acquisitions with the following parameters: TR = 822 ms (9.4 T) or 812 ms (14.1 T), AD = 1.3 ms, flip angle (FA) = 55° (9.4 T) or 52° (14.1 T), BW = 5000 Hz (9.4 T) or 7143 Hz (14.1 T), 768 (9.4 T) or 1024 (14.1 T) spectral points, 31 × 31 matrix, FOV = 24 × 24 mm^2^, voxel size = 0.77 × 0.77 × 2 mm^3^, single average, water suppression using variable power radiofrequency pulses and optimized relaxation delays (VAPOR) scheme, lipid suppression using 12 (9.4 T) or six (14.1 T) saturation bands placed on cranial regions. Water data were acquired using the same parameters, except that water suppression was disabled.

##### SE-MRSI

Metabolite and water data were acquired from the same experimental setup procedure and scanning parameters as used in FID-MRSI at 9.4 T, except that the SE-MRSI was used at 9.4 T with TR = 2000 ms, TE = 3.3 ms, FA = 90°/180°.

More detailed methods and scanning parameters for MRSI data can be found in our other studies [6, 32, 44].

#### SVS data

##### STEAM SVS

Metabolite data were acquired from a 2.8 × 2 × 2 mm^3^ voxel in the hippocampus. STEAM was used at 9.4 T with the following parameters: TR = 4000 ms, TE = 3 ms, TM = 10 ms, BW = 5000 Hz, 4096 spectral points, 16 averages, 16 repetitions, and water suppression using VAPOR. Water data were acquired using the same parameters, except that water suppression was disabled.

##### SPECIAL SVS

Metabolite data were acquired from a 2.8 × 2 × 2 mm^3^ voxel in the hippocampus. SPECIAL [36] was used at 14.1 T with the following parameters: TR = 4000 ms, TE = 9.3 ms, BW = 7143 Hz, 4096 spectral points, 256 repetitions, water suppression using VAPOR, and an additional water suppression pulse inserted in the mixing time (TM). Water data were acquired using the same parameters, except that water suppression was disabled.

#### DWS data

Metabolite data were acquired from a 10 × 2.5 × 4 mm^3^ (9.4 T) or 7 × 5 × 5 mm^3^ (14.1 T) voxel, mostly covering the hippocampus. dSPECIAL [37] was used at 9.4 T or 14.1 T with the following parameters: TR = 3000 ms, TE = 9.5 ms, TM = 150 ms (9.4 T) or 40 ms (14.1 T), BW = 5000 Hz (9.4 T) or 7143 Hz (14.1 T), 4096 spectral points, b-value = 0.05, 1.5, 4, 8, 15, 22, 30 ms/µm^2^ with 64, 64, 96, 96, 128, 128, 192 repetitions, respectively (9.4 T), or 0.05, 1, 3, 5, 10, 20 ms/µm^2^ with 160, 160, 160, 160, 320, 320 repetitions, respectively (14.1 T), and water suppression using VAPOR and an additional water suppression pulse in TM.

#### Basis set

Basis sets were generated using metabolites simulated by NMRScopeB from jMRUI [45–47], employing the same acquisition sequence and parameters as the corresponding in vivo measurements and the experimentally measured macromolecule signal under the same conditions [6, 30, 32, 37, 43]. Simulations incorporated published J-coupling constants and chemical shifts [48–50]. Each basis set was tailored to the specific scan configuration of the dataset, including sequence type, TE, AD, magnetic field strength, and other relevant parameters.

## Results

### MRSI data from different sequences and field strengths

**Fig. 5:**
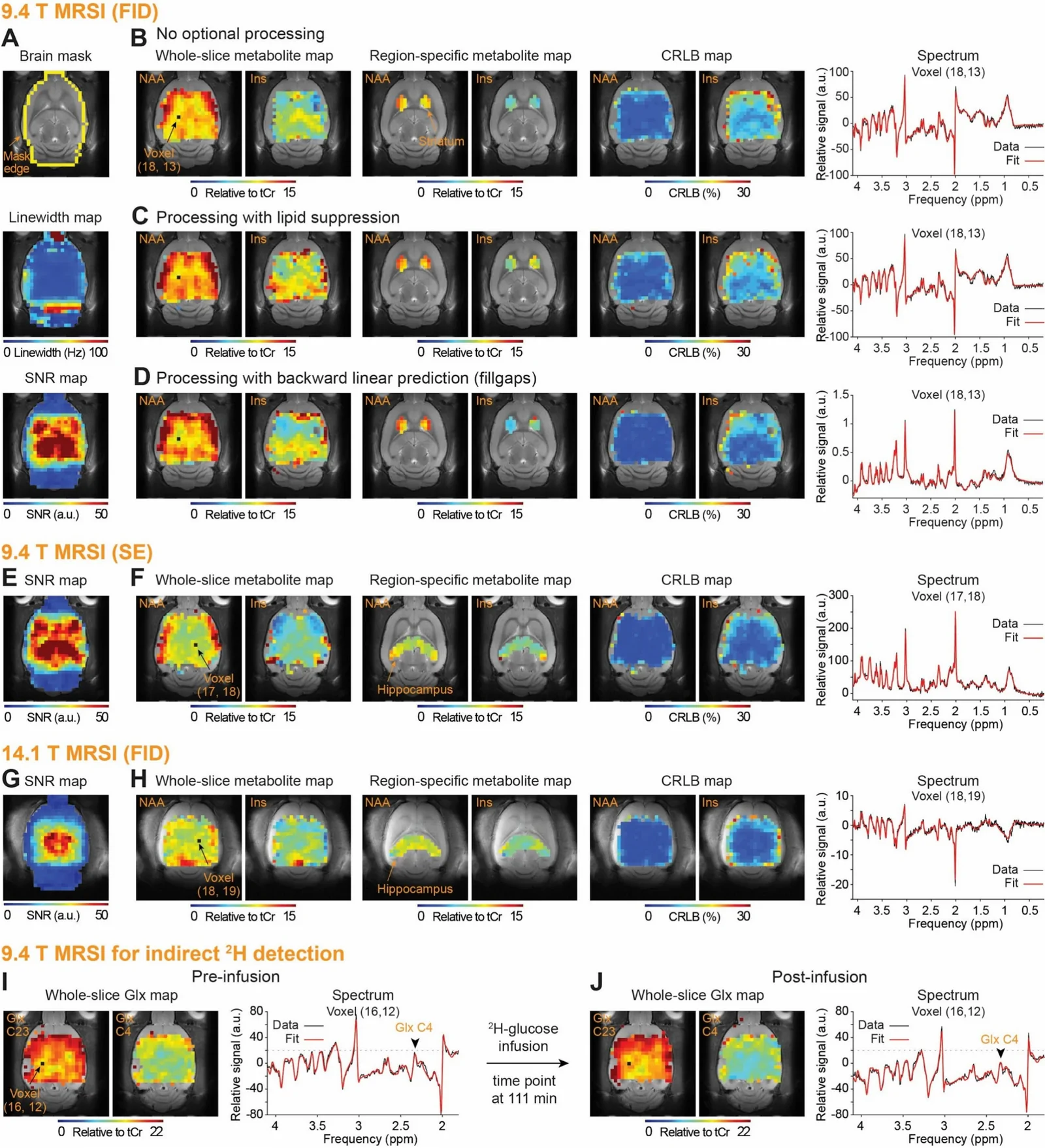
Results from processing MRSI demonstration datasets. **A–D** FID-MRSI data at 9.4 T. **A** Top to bottom: brain mask output from the co-registration step, linewidth map from water data, and SNR map from metabolite data after applying the brain mask. **B** Processing with default options. Left to right: whole-slice and region-specific (striatum) metabolite maps, CRLB maps for NAA and Ins, and fitted spectrum (red) overlaid on data from a representative voxel (black). **C** The same as (**B**), but for processing data with an additional lipid suppression option. **D** The same as (**B**), but for processing data with an additional backward linear prediction (fillgaps) option. **E** and **F** SE-MRSI data at 9.4 T. **E** SNR map from metabolite data after applying the brain mask. **F** Processing with default options. Left to right: whole-slice and region-specific (hippocampus) metabolite maps, CRLB maps for NAA and Ins, fitted spectrum (red) overlaid on data from a representative voxel (black). **G** and **H** The same as (E and F), but for FID-MRSI data at 14.1 T. **I** and **J** Indirect ^2^H-FID-MRSI data at 9.4 T detecting the expected Glx-C4 ^1^H signal decay induced by Glx ^2^H-labeling from local metabolism of the infused [6,6’-^2^H₂] Glc. **I** Metabolite maps (left) acquired before a ^2^H-Glc infusion protocol (2 h) showing Glx-C23 and Glx-C4 ^1^H signals as detected by LCModel fitting of ^1^H-FID-MRSI spectra (right). **J** Metabolite maps (left) acquired post ^2^H-Glc infusion protocol (last time point, i.e., 111 min after the infusion started) showing Glx-C23 and Glx-C4 ^1^H signal as detected by LCModel fitting of ^1^H-FID-MRSI spectra (right). Quantifying the relative decay of Glx-C4 ^1^H signal for each FID-MRSI iteration with respect to the acquired baseline (**I**) led to the estimation of ^2^H-Glx turnover along the 2-h ^2^H-Glc infusion

Following the described practical guidelines, we first demonstrate the MRSI functionalities using the options available in the MRSI processing pipeline. There are four datasets from different sequences and field strengths used for this purpose: FID-MRSI data at 9.4 T (Fig. 5A–D), SE-MRSI data at 9.4 T (Fig. 5E and F), FID-MRSI data at 14.1 T (Fig. 5G and H), and indirect ^2^H-FID-MRSI data at 9.4 T (Fig. 5I and J). For FID-MRSI, Fig. 5A shows brain mask edges overlaid on the anatomical image, obtained via co-registration between the acquired MRI image and the anatomical template (Fig. 5A, top). Similarly, the linewidth map (Fig. 5A, middle) estimated from water MRSI data and SNR maps calculated using metabolite MRSI data (more specifically NAA) after applying the brain mask (Fig. 5A, bottom) are shown as outputs from the toolbox. With the default processing option, we obtained NAA and myo-inositol (Ins) metabolite maps for the whole-brain slice, and with regional highlights such as the striatum delineated by brain segmentations. In addition, CRLB maps extracted from LCModel outputs were included to assess the fitting accuracy, as illustrated in the first six columns of Fig. 5B. These results from the metabolite map window were simply exported using the *Save map* button. As another output of the toolbox, the spectrum for a voxel selected in the *Spectral display* window is presented in the last column of Fig. 5B. The result can also be quantitatively presented using the *Concentration table* (Supplementary Table 3) and, in this case, tCr as internal reference, displaying averaged values from counted voxels in the hippocampus. To demonstrate the capability of the preprocessing options, we repeated the analyses, but processing with *lipid suppression* (Fig. 5C) and with *Fillgaps* (Fig. 5D). Slight changes in the maps upon applying the preprocessing steps were observed (Fig. 5C and D). In results from processing with *Fillgaps*, the output provides fully phased spectra, indicating the effects of backward linear prediction of missing points in FID (see the last column of Fig. 5D).

For other sequences, the toolbox produced excellent maps and spectra, as shown in Fig. 5E and F for SE-MRSI at 9.4 T, and Fig. 5G and H for FID-MRSI at 14.1 T, demonstrating the capability of the *MRS4Brain Toolbox* to process MRSI data across a broad range of dataset types.

The toolbox’s MRSI functionalities can also process indirect ^2^H-FID-MRSI data (Fig. 5I and J). These data were acquired before and during a 2-h ^2^H-Glc infusion protocol via 2D ^1^H-FID-MRSI iterations (acquisition time = 13 min, see Demonstration data acquisition for details), to quantify the progressive ^2^H-labeling of Glx (glutamine and glutamate) at its position C4 in the rat brain via quantification of the respective ^1^H signal decay [43]. While the Glx-C23 ^1^H spectral signal appeared stable in the comparison between pre- and post-infusion metabolite maps (Fig. 5I and J, respectively), the Glx-C4 ^1^H signal (2.3 to 2.4 ppm) showed a significant and generalized decay in the MRSI slice, as detected by LCModel fitting of ^1^H-FID-MRSI spectra. The quantification of this ^2^H-Glx 2-h turnover following a controlled ^2^H-Glc infusion protocol, in combination with the proper metabolic modeling scheme, can provide a kinetic characterization of the oxidative pathway of glucose metabolism in the brain, broadening the application possibilities of MRSI from a static characterization to a dynamic analysis of metabolism [43].

Voxels in the metabolite maps passed the quality control thresholds as SNR ≥ 10, linewidth ≤ 1.25 times the averaged linewidth, and CRLB < 30%. Metabolite maps are presented relative to tCr fixed at 8 mmol/kg_ww_. Black dots in the NAA map indicate voxel locations for the representative spectra in the last column.

### SVS data from different sequences and field strengths

**Fig. 6:**
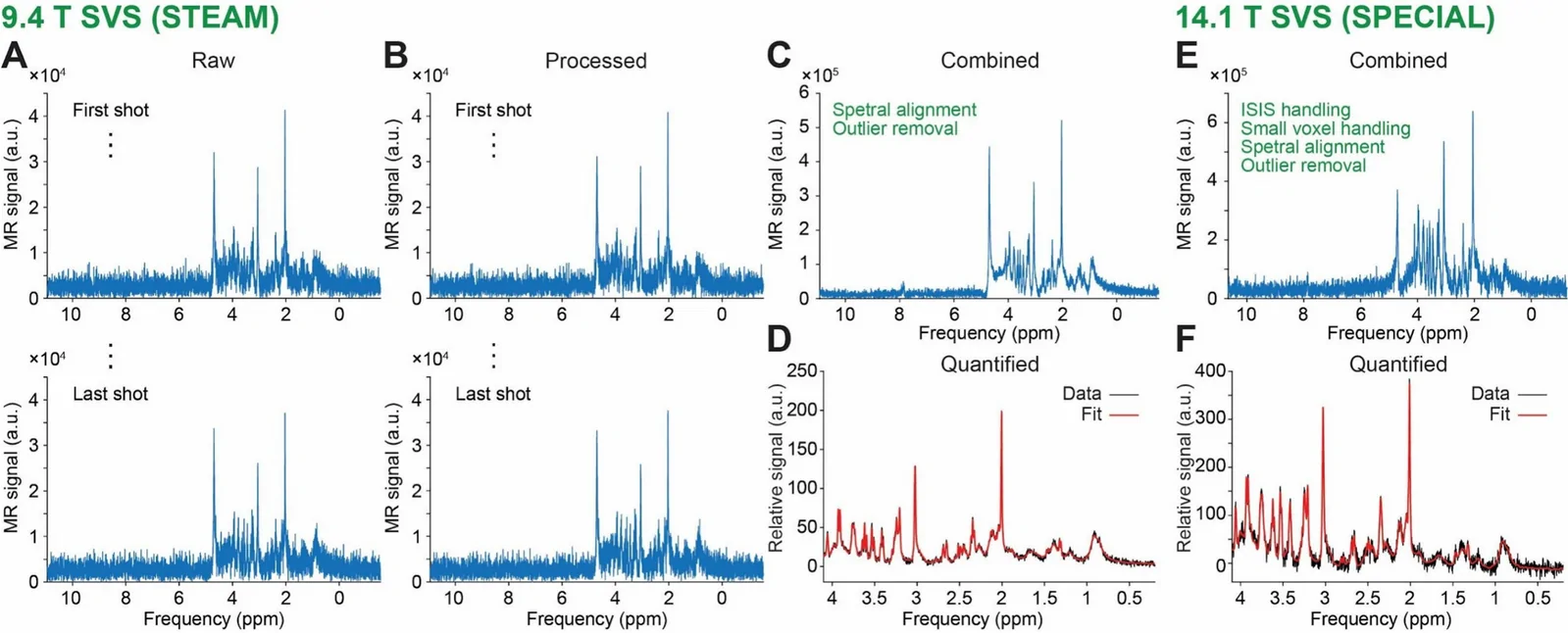
Results from processing SVS demonstration datasets. **A–D** STEAM SVS data at 9.4 T. **A** Unprocessed (raw) spectrum displayed individually from the first shot (top) to the last one (bottom), after selecting *Choose selection*. **B** The same as (**A**), but after applying spectral alignment. **C** Spectrum with all shots combined, excluding outliers. **D** LCModel’s fitted spectrum (red) displayed with data (black). **E** and **F** SPECIAL data at 14.1 T. The same as **C** and **D** but processed with additional preprocessing steps such as small voxel and ISIS handling

Next, we examined the SVS functionalities using the available options in the SVS processing pipeline. Two datasets acquired in the hippocampus using different sequences and field strengths were examined: STEAM data at 9.4 T (Fig. 6A to D) and SPECIAL data at 14.1 T (Fig. 6E and F). For the STEAM dataset, selecting the *Choose Selection* option displayed the raw spectra, allowing users to inspect individual spectra from the first to the last shot (Fig. 6A, top and bottom, respectively). Upon selecting the *Data processing* button, the pipeline generated both *processed* spectra (Fig. 6B) and *combined* spectra (Fig. 6C). By default, the *combined* spectrum, including *Spectral alignment* and *Outlier removal*, was exhibited. *Processed* spectra for each shot (Fig. 6B) could be viewed by selecting *processed* in the *Type* dropdown menu. Following LCModel fitting, the *quantified* spectrum was shown with the measured data in black and the fitted one in red (Fig. 6D). Also, a results table was generated, listing metabolite names, LCModel relative and “absolute” concentrations, and CRLB values (Supplementary Table 4). Processing of the SPECIAL dataset followed the same general procedure as used in STEAM data analysis. However, because the data were acquired using an ISIS localization scheme, ISIS handling was required, in addition to Spectral Alignment and Outlier Removal. Small voxel handling was also enabled for this demonstration (Fig. 6E). The final output showed the fitted LCModel spectrum overlaid on the measured data (Fig. 6F), confirming successful analysis of the SPECIAL dataset.

Overall, these results demonstrate that the SVS processing pipeline can reliably process SVS datasets acquired with different sequences and at different field strengths using the full suite of functionalities available in the toolbox.

### DWS data at different field strengths

**Fig. 7:**
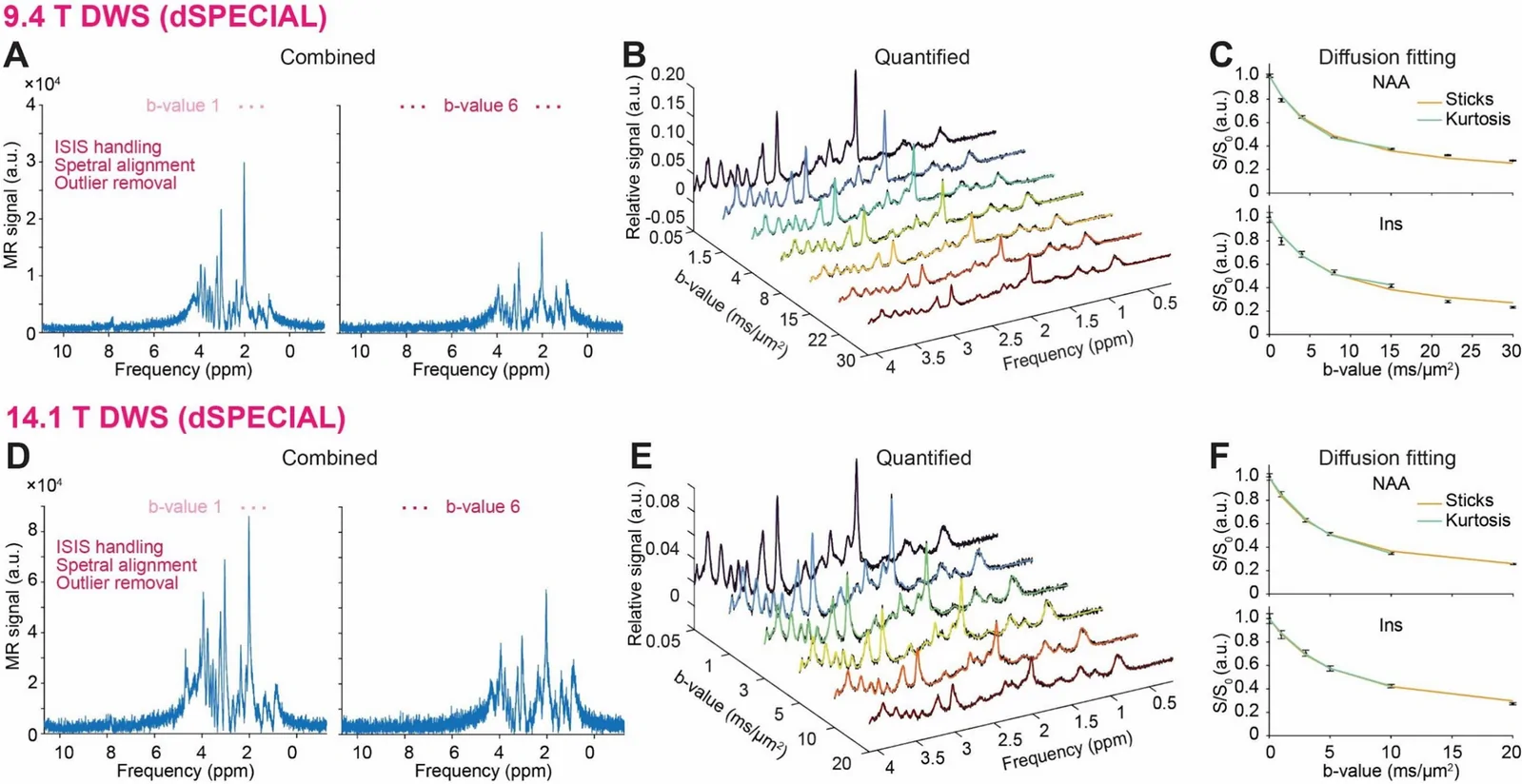
Results from processing DWS demonstration datasets **A–C** dSPECIAL DWS data at 9.4 T. **A** Spectrum obtained by combining all shots, following processing using ISIS handling, spectral alignment, and outlier removal, for individual datasets: b-value 1 (left) and b-value 6 (right). **B** Spectra across all b-values displayed in 3D plotting mode, with LCModel’s fitted spectra (rainbow colors) overlaid on the data (black). **C** Diffusion fits using randomly oriented sticks (yellow) and kurtosis (green) models for NAA (top) and Ins (bottom), plotted as a function of b-values. **D–F** The same as **A**–**C**, but for dSPECIAL DWS data at 14.1 T

Finally, we evaluated the DWS functionalities using the available options in the DWS processing pipeline. Two datasets acquired in the hippocampus with the dSPECIAL sequence at different field strengths were analyzed: 9.4 T (Fig. 7A to C) and 14.1 T (Fig. 7D to F). Similar to the SVS processing pipeline, spectra for each average/shot, either *raw* or *processed*, are displayed in the *Spectral display* window once each processing step is completed. Because the dSPECIAL data were acquired using an ISIS localization scheme, *ISIS handling* was required in addition to *Spectral alignment* and *Outlier removal*. After preprocessing, *combined* spectra for each b-value were generated (Fig. 7A and D). Selecting a dataset from the *Experiment* dropdown menu allows users to inspect the spectrum for a given b-value and assess preprocessing effects. Quantification is performed by selecting the *Quantification* button, which generates a fitted spectrum overlaid with the processed data, consistent with the SVS pipeline. Unique to the DWS pipeline, the *3D plot* (Fig. 7B and E) provides an efficient overview of all spectra within a single frame. This enables a quick evaluation of diffusion-related signal attenuation across b-values, even when datasets across b-values were acquired in random order, as data indices can be rearranged using the *Data index* box. In addition to a table summarizing concentration or arbitrary unit values (Supplementary Table 5), the toolbox offers *Diffusion fitting*, which displays the relative signal, S/S₀, computed as the signal at a given b-value normalized to the signal at the smallest b-value. In this demonstration, DWS data were fitted using either an empirical model (e.g., kurtosis) or a simplified biophysical model (e.g., the commonly used randomly oriented stick model) (Fig. 7C and F). Estimated parameters, including D_intra_, ADC, and AKC, are displayed as shown in Fig. 4B (left). Because each model is valid over a specific b-value range, the toolbox allows users to select an appropriate fitting range. For the kurtosis model, suitable b-values typically extend up to 10—15 ms/µm^2^, depending on the metabolites [51].

Together, these results demonstrate that the DWS processing pipeline can robustly process various types of DWS datasets (e.g., dSPECIAL acquired at different field strengths) using the full set of functionalities implemented in the toolbox.

## Discussion

We developed and presented herein the *MRS4Brain Toolbox*, a freely available software to streamline and facilitate the processing of preclinical ^1^H-MRSI and indirect ^2^H-MRSI, MRS, and DWS datasets, even for non-spectroscopy experts. This comprehensive tool combines processing pipelines to process datasets acquired on preclinical Bruker scanners. For proton and deuterium-based MRSI, it enables automated processing with semi-automatic quality control, removal of spurious signals, spectral fitting, brain segmentation, visualization, and co-registration of the metabolic maps, and built-in statistical tools. This allows streamlined processing of the huge amount of data generated in MRSI acquisitions, which would not be feasible manually in a reliable manner. Covering a comprehensive set of spectroscopy methods, it also includes pipelines for preprocessing and quantification of single-voxel spectroscopy datasets, as well as fitting of diffusion-weighted spectroscopy, allowing for a variety of uses for preclinical spectroscopic studies.

In the current implementation, the *MRS4Brain Toolbox* is compatible with datasets acquired on Bruker systems running ParaVision 360, which have been installed since 2019. As such, usage of the toolbox is limited to the data format predefined by the ParaVision software. One possibility to explore is the ability to read data in a MATLAB format, which would require the user to have their own data reading code to translate from any vendor format to a readable MATLAB file that could skip the reading step of the process. Additionally, while the toolbox manages to cover a variety of sequences, there exist some blind spots for advanced MRSI sequences, such as spatial-spectral encoding MRSI. Indeed, as the *MRS4Brain Toolbox* is designed to work with vendor-provided sequences to facilitate its use on many sites without having to program or install custom acquisition sequences, it only supports datasets that are already spatially reconstructed (in image space). To cover this issue, we plan to use the *rawdata.job0* file provided by ParaVision 360 containing the raw *k*-space data acquired, and to proceed to the sequence-specific reconstruction to fall back into a data structure more akin to what is found in *.fid or *.fid_proc.64. This future work may be implemented for both fully sampled and undersampled MRSI datasets. Undersampled datasets may further benefit from advanced compressed sensing reconstruction approaches such as low-rank total generalized variation (LR-TGV) [7, 52] or iterative self-consistent parallel imaging reconstruction (SPIRiT) [53]. All these developments would be beneficial to accelerate both proton and deuterium-based MRSI acquisitions. We have recently shown promising results using different denoising strategies for both proton and deuterium-based datasets, MRSI and DWS, e.g., Marchenko–Pastur principal component analysis (MP-PCA), total variation (TV) regularization [7, 54, 55], stacked partial separability and spectral and temporal integration for SVD (SPIN-SVD), and tensor MP-PCA (tMPPCA) [56, 57]. Next versions of the *MRS4Brain Toolbox* will integrate these denoising strategies to support robust, automated workflows across a wide range of preclinical MR spectroscopy applications, with particular benefit for X-nuclei MRSI, where an inherently lower signal-to-noise ratio poses significant challenges. In addition, we will incorporate thorough testing and validation of X-nuclei MRSI and MRS data sets combined with advanced metabolic modeling, e.g., for both direct and indirect ^2^H kinetic analysis [43], as recently demonstrated by our group. For fit quality assessment, the fit quality number (FQN) would be a valuable additional metric for evaluating the quality of model fits [17], capturing aspects of spectral quality that may not be fully reflected by CRLB alone. Therefore, the implementation and validation of FQN are planned for future versions of the toolbox.

Although architecturally similar to the MRS processing toolbox for human data, the presented *MRS4Brain Toolbox* fills a gap in preclinical metabolic imaging research by providing a pipeline tailored to the specificity of preclinical MRS and MRSI, beyond vendor-specific formats. In particular, the relatively low SNR related to the small size of the rodent brain, the typically larger partial volume of muscle, skin, and fat in the field of view of the radiofrequency coil, the shimming of large volumes with many tissue interfaces and the limited measurable matrix size are typical differences compared to human MRSI data, for which the *MRS4Brain Toolbox* is optimized. Registration of preclinical images acquired with lower matrix sizes and typically less homogeneous B_1_, as in humans, is also integrated with corresponding brain atlases.

The implementation of advanced sequences can be extended to DWS since most sequences related to this modality used in research are custom-built. As it stands, the toolbox is optimized for sequences using a single b-value per acquisition. The toolbox may require adaptation for specific sequence implementations, such as those employing interleaved b-values and/or gradient directions within a single acquisition protocol [58, 59]. Including a monoexponential model would be useful for datasets with only a few small b-values, or including a rapid quality check to verify data consistency, consisting of spectra displays for each processing step. Beyond this, incorporating more sophisticated models would be highly beneficial for further applications and could help democratize the adoption of DWS by enabling the estimation of microstructural parameters such as soma radius and projection radius, as in the soma and neurite density imaging (SANDI) model [60]. These advanced models are particularly relevant because they allow moving beyond simple diffusion metrics to infer biologically meaningful features of tissue microstructure, such as cell body size and neurite geometry, which are critical for understanding brain organization and pathology. Models such as sphere-and-stick or cylinder could also be integrated to broaden the toolbox’s modeling capabilities and support more detailed microstructural analysis. Finally, processing an analysis that include multiple diffusion times would also be in line with recent publications [61, 62], offering more potential applications for DWS in preclinical research. In upcoming versions of the toolbox, additional functionality will likely include advanced visualization tools for data processing steps. This would be particularly important to align with best practices in DWS [40], which require extensive quality checks since even minor animal movements can significantly affect the DWS signal. For example, useful visualization features could include overlaying individual repetitions to verify alignment and tools for outlier detection and removal, with an option to manually select specific outliers for exclusion.

Efficient brain function depends on the seamless integration of localized neural processing with long-range network connectivity, in which focal activations support specialized functions and long-range connections facilitate efficient information exchange between distributed brain regions. Together, these processes underpin normal and efficient cognitive functioning, sensory processing, motor control, and other behavioral operations. In light of this network-based perspective, the long-term trajectory of preclinical MRSI research is expected to move beyond characterizing region-specific alterations but toward characterizing the brain as an integrated network to better capture the diverse pathological processes associated with neurological disease [63, 64]. To support this shift, one of our aims is to extend the current analysis toolbox with a new feature capable of generating metabolic similarity matrices, enabling the evaluation of metabolic relationships across brain regions and advancing the study of network-level metabolic dysfunction.

The same atlas-based architecture that supports metabolic maps and connectivity analyses in MRSI can be extended to positron emission tomography (PET) studies, providing a natural multimodal bridge toward PET-derived metabolic flux maps, as can be obtained, for example, with fluorodeoxyglucose (FDG)-PET [65], or metabolic connectivity in future implementations. PET represents a mature molecular imaging modality capable of quantifying pathway-specific metabolism and receptor-level binding affinity with high sensitivity, and its integration constitutes a logical expansion of the toolbox toward broader multimodal characterization of healthy brain function and pathologies. Recent work in rodent imaging has shown that reproducible tracer-derived biomarkers require MRI-based co-registration and atlas-defined regional sampling to ensure anatomical precision and reduce methodological variability [66]. In PET images, anatomical reference points are typically missing, with the PET intensity distribution reflecting tracer-specific molecular fate rather than underlying tissue structure, and some tracers show no uptake in major brain regions. Multimodal PET–computed tomography (CT) has become a standard multimodal approach to obtain fast anatomical reference images. However, PET–MRI offers superior soft tissue contrast, in particular in the brain, with better tissue differentiation and without the use of additional ionizing radiation. Moreover, the use of MRI equipment opens the way to all MR-based approaches, such as functional MRI for functional studies and MRSI for metabolic imaging. Within this framework, PET and MRSI provide complementary readouts: PET quantifies molecular kinetics (e.g., receptor binding or glucose utilization flux with FDG-PET), whereas MRSI reflects the intracellular biochemical state, its integrity, or perturbation in specific brain diseases. These coordinated measurements have shown their potential in rodent disease models, such as bile duct-ligated (BDL) rats for the study of hepatic encephalopathy, where regional reductions in glucose metabolism, measured with FDG-PET, were shown to co-occur with perturbations in glutamatergic and osmotic metabolites detected by ^1^H-MRS [65]. Beyond such biological complementarity, recent methodological developments have demonstrated that dynamic FDG-PET can also be used to derive within-individual metabolic connectivity networks [67], providing metabolic information beyond static regional measurements. Together, these findings highlight that both MRSI and single-voxel spectroscopy can provide metabolic information that meaningfully complements PET-derived measures and therefore represent valuable approaches for future multimodal PET–MR integration. Future releases of the *MRS4Brain Toolbox* will incorporate PET processing by leveraging the existing ANTs-based registration, atlas segmentation, and regional statistical framework, enabling PET–MRI-atlas alignment and spatially resolved multimodal analyses with MRSI/MRS-derived metabolic profiles.

## Conclusion

We introduced the *MRS4Brain Toolbox*, a freely available and comprehensive software designed to streamline the processing of preclinical ^1^H-MRSI and indirect ^2^H-MRSI, MRS, and DWS datasets via a graphical user interface, even for non-experts in spectroscopy. By integrating automated and semi-automatic pipelines for preclinical acquisitions such as Bruker data, the toolbox enables efficient handling of large-scale MRSI data through robust quality control, different processing steps, spectral fitting, brain segmentation, visualization, metabolic maps, and statistical analysis. Support for both SVS and DWS, including modeling, further extends its capability across a wide range of preclinical studies. By reducing manual workload and ensuring reproducibility, *MRS4Brain Toolbox* facilitates basic and advanced preclinical MR spectroscopic research and promotes broader adoption of ^1^H and X-nuclei MRSI, MRS, DWS, and multimodal bioimaging methodologies in neuroscience.

## Supplementary Information

Below is the link to the electronic supplementary material.

Supplementary file1 (DOCX 3814 KB)

## Data Availability

The current version of the *MRS4Brain Toolbox* and the datasets for demonstration purposes are publicly available on the GitHub [23], Zenodo [24] repositories, and our MRS4Brain group’s website (https://www.epfl.ch/labs/mrs4brain/ressources/mrs4brain-toolbox/).
