# Supplementary material for: MRS4Brain: a software for preclinical proton and deuterium-based MR spectroscopic imaging data
Source: MAGMA. 2026 Apr 8;39(3):361–79. doi: 10.1007/s10334-026-01351-w (PMC13687256; doi:10.1007/s10334-026-01351-w)
Supplement: Supplementary file 1 [file 10334_2026_1351_MOESM1_ESM.docx]

**Supplementary Information**

**MRS4Brain: a software for preclinical proton and deuterium-based MR spectroscopic imaging data**

Brayan Alves^1,2,3†^, Tan Toi Phan^1,2,3†*^, Guillaume Briand^1,2,3^, Alessio Siviglia^1,2,3^, Gianna Nossa^1,2,3^, Jessie Mosso^1,2,3,4^, Eloïse Mougel^1,2,3^, Jamie Near^5,6^, Thi Ngoc Anh Dinh^1,2,3^, Omar Zenteno^1,2,3^, Bernard Lanz^1,2,3^, Thanh Phong Lê^1,2,3*^, Cristina Cudalbu^1,2,3*^

^1^CIBM Center for Biomedical Imaging, Lausanne, Switzerland

^2^CIBM Pre-Clinical Imaging Section, EPFL - École Polytechnique Fédérale de Lausanne, Lausanne, Switzerland

^3^MRS4Brain Group, School of Basic Sciences, EPFL - École Polytechnique Fédérale de Lausanne, Lausanne, Switzerland

^4^Bernard and Irene Schwartz Center for Biomedical Imaging, Department of Radiology, New York University Grossman School of Medicine, New York, NY, USA.

^5^Physical Sciences, Sunnybrook Research Institute, Toronto, Ontario, Canada

^6^Department of Medical Biophysics, Temerty Faculty of Medicine, University of Toronto, Toronto, Ontario, Canada

† Brayan Alves and Tan Toi Phan have contributed equally to this work.

* Correspondence: Cristina Cudalbu (cristina.cudalbu@epfl.ch), Tan Toi Phan (tantoi.phan@epfl.ch), and Thanh Phong Lê (thanh.le@epfl.ch)

**Installation guideline**

The *MRS4Brain Toolbox* is compatible with MATLAB 2023a or later on all major operating systems. Three MATLAB add-on toolboxes are required to execute the toolbox’s processing pipelines: the Image Processing Toolbox (version 11.7 or newer), the Signal Processing Toolbox (version 9.2 or newer), the Parallel Computing Toolbox (version 7.8 or newer), and the Statistics and Machine Learning Toolbox (version 12.5 or newer).

In addition to MATLAB dependencies, four external software packages are necessary for full toolbox functionality: Advanced Normalization Tools (ANTs) for MRI co-registration, Docker to support the ANTs installation, LCModel for spectral fitting and quantification, and FID-A for specific preprocessing steps within the SVS pipeline. The FID-A functionalities required by the toolbox are already implemented internally, and therefore, no installation of FID-A is needed. Depending on the operating system, ANTsX/ANTs may require system-specific installation procedures for use in the MRI co-registration workflow. To streamline installation across platforms, including Windows and macOS, a Docker container (https://www.docker.com/) is employed. When the toolbox is used for the first time, if Docker is running, the toolbox automatically pulls the ANTs image into Docker. Users may alternatively download the image manually via Docker by searching for *antsx/ants:v2.5.0*. Download and installation may require several minutes to complete. Instructions for obtaining and installing LCModel are available on the LCModel website (https://s-provencher.com/lcmodel.shtml). Windows/macOS binaries are also provided in a GitHub repository with detailed installation guidance (https://github.com/schorschinho/LCModel).

At each new MATLAB session, directories for the toolbox folder and all its subfolders must be added to the MATLAB path. This can be accomplished by right-clicking the toolbox folder in MATLAB’s Current Folder panel and selecting *Add to Path*, then *Selected Folders and Subfolders*, or by using the *genpath()* and *addpath()* commands in the Command Window. The toolbox is launched by entering *MRS4BRAIN_toolbox* in the MATLAB Command Window.

**Descriptions for data quality metrics used in Quality control**

SNR: The SNR is computed in the frequency domain, using the NAA peak at 2.01 ppm. The formula is as follows: $SNR=\frac{A_{NAA}}{\sigma_{Noise}}$

Where $A_{NAA}$ is the amplitude of the NAA peak in absolute mode, and $\sigma_{Noise}$ is the standard deviation of the signal measured in a metabolite-free region (between 0.5 and -0.5 ppm in this manuscript).

Linewidth: The linewidth is measured from the water peak. At first, the real water spectrum is 0-order phase-corrected to ensure that the peak is entirely in the absorption mode. Once phase corrected, the 4 spectral points closest to 50% of the real water peak amplitude, 2 left-side and 2 right-side, are located, and their corresponding frequencies are stored. Each pair is then used to create a linear interpolation to determine the two frequencies (minimum and maximum) at which we reach 50% of the real water peak amplitude. The difference between these two frequencies gives us the water linewidth. As the metabolite linewidths are smaller than water due to differences in T2*, the water linewidth provided with the toolbox serves as a supremum of the linewidth measured within a metabolite measurement.

CRLB: The Cramér-Rao lower bounds used for quality control are provided as LCModel output. In that case, the CRLB is viewed as the minimal theoretical uncertainty of the model obtained with LCModel fitting. This metric should be evaluated and interpreted with care; more in-depth information on its use and interpretation can be found in the reference cited in the main manuscript.

**List of statistical tests in the Statistical tool for MRSI**

- Student’s t‑test, used for comparing two results within subjects (paired) or between subjects (unpaired), depending on the experimental design.

- One-way analysis of variance (ANOVA) with Bonferroni's multiple comparisons, possibly used for comparing three or more results when there is one independent factor, such as subject group, brain region, or analysis type.

- Two-way ANOVA with Bonferroni's multiple comparisons, possibly used for comparing three or more results across two independent factors, such as subject group × brain region, or analysis type × brain region, typically between subjects.

Options: All tests may be performed with one‑sided or two‑sided p‑values, depending on the hypothesis.

It is essential to carefully select statistical tests and analysis options, as the appropriate choice depends on the experimental design and the underlying study hypothesis.

**Figure S1. Statistical tool for MRSI outputs.** Practical steps from importing results, selecting metabolites, the testing type, and its parameters, to performing a statistical analysis with graphs, *p-*values, and statistical values (e.g., *t-*value) provided for MRSI outputs.

**Supplementary Table 1. List of user interface components in MRSI, SVS, and DWS windows**

| **Window** | **Components** | **Descriptions** |
| --- | --- | --- |
| MRSI | ***Data management*** panel |  |
|  | *Bruker data folder* button | To specify the input data directory |
|  | *Result folder* button | To specify the output result directory |
|  | *Study name* box | To define a unique output folder name for each analysis |
|  | *Red folder* button | To select preexisting output result directory (overwrites the content of *Study name*) |
|  | ***Processing steps*** panel |  |
|  | *3D MRSI* checkbox | To activate 3D/multi-slice data handling |
|  | *Number slices* spinner | To specify the number of slices used for 3D/multi-slice data analysis |
|  | *Lipid suppression* button | To activate the lipid suppression feature |
|  | *Fillgaps* button | To activate the fillgaps feature |
|  | ***Data specific*** panel |  |
|  | *PDF document* button | To open the scan note PDF if needed |
|  | *Head prone* checkbox | To adjust image orientation if the head-prone is used during acquisition |
|  | *Reco Nb* box | To specify Bruker’s reconstruction number |
|  | *Metabolite* box | To specify the scan number for metabolite data |
|  | *Ref* box | To specify the scan number for water data |
|  | *Ref* checkbox | To activate if using water data as a reference |
|  | *Image* box | To specify the scan number for the anatomical image |
|  | *Slice range* box | To specify the number of slices combined for brain segmentation |
|  | *MRI slice* table | To list the slice numbers for analysis and display |
|  | ***Options*** panel |  |
|  | *MRSI parameters* button | To define the atlas and LCModel fitting configurations |
|  | *Statistical tool* button | To open the statistical tool window |
|  | ***Show Results*** panel |  |
|  | *Slice* spinners | To select the slice number for outputs if multi-slice data are analyzed |
|  | *Custom regions* checkbox | To activate the feature of defining the custom regions |
|  | *Region* dropdown menu | To select the brain region name for display and outputs |
|  | *Metabolite* dropdown menu | To select the metabolite name or type of mask for display and outputs |
|  | *Side* dropdown menu | To select the brain side for display and outputs |
|  | *Concentration* dropdown menu | To select the concentration type from the LCModel outputs |
|  | *Overlap* slider | To control the voxel overlap ratio in resampling the atlas to MRSI resolution |
|  | *Brain volumetry table* button | To open the table of subject brain volumetry compared with the atlas |
|  | *Manual voxel selector* button | To open the window for user-defined regions-of-interest |
|  | *Concentration table* button | To open the table of metabolite concentration |
|  | *Quality control* button | To open the window for adjusting the map appearance |
|  | *Spectral display* button | To open the window for showing a spectrum from a voxel of interest |
|  | *Save map* button | To save the map/mask currently displayed in the window |
|  | *MRSI Analysis* button | To execute the analysis |
| SVS | ***Data management*** panel |  |
|  | *Result folder* button | To specify the output result directory |
|  | *Study name* box | To define a unique output folder name for each analysis |
|  | *Red folder* button | To select preexisting output result directory (overwrites the content of *Study name*) |
|  | *Load SVS data* button | To open the window for loading input data |
|  | ***Experiments*** panel |  |
|  | *Reference data* dropdown menu | To select a dataset for reference |
|  | *Metabolite data* table | To select datasets for analysis |
|  | *Select all* button | To select all datasets in the list |
|  | *Deselect all* button | To deselect all datasets in the list |
|  | *Choose selection* button | To read selected datasets |
|  | *Data Processing* button | To perform processing steps |
|  | *Quantification* button | To perform LCModel fitting and quantification |
|  | *SVS Parameters* button | To define LCModel fitting configurations and preprocessing steps |
|  | *Shot selector* buttons | To select the uncombined shot/repetition/average number for display |
|  | *Experiment* dropdown menu | To select the result name for display and outputs |
|  | *SNR* box | To display the calculated signal-to-noise ratio (SNR) |
|  | *Scale Min* & *Max* boxes | To adjust the y-axis scale if needed |
|  | *Type* dropdown menu | To select the type of data/results for display and outputs |
|  | *abs* button | To display the absolute amplitude of signals |
|  | *real* button | To display the real part of the signals |
|  | *imag* button | To display the imaginary part of signals |
|  | *Data* button | To display the SVS experimental spectrum |
|  | *Fit* button | To display the LCModel fitting |
|  | *Baseline* button | To display the LCModel baseline |
|  | *Concentration* table | To show the concentration from the LCModel outputs |
| DWS | ***Data management*** panel |  |
|  | *Result folder* button | To specify the output result directory |
|  | *Study name* box | To define a unique output folder name for each analysis |
|  | *Red folder* button | To select preexisting output result directory (overwrites the content of *Study name*) |
|  | *Load DWS data* button | To open the window for loading input data |
|  | ***Experiments*** panel |  |
|  | *Reference data* dropdown menu | To select a dataset for reference |
|  | *Metabolite data* table | To select datasets for analysis |
|  | *Select all* button | To select all datasets in the list |
|  | *Deselect all* button | To deselect all datasets in the list |
|  | *Choose selection* button | To read selected datasets |
|  | *Data Processing* button | To perform processing steps |
|  | *Quantification* button | To perform LCModel fitting and quantification |
|  | *DWS Parameters* button | To define LCModel fitting configurations and preprocessing steps |
|  | *Shot selector* buttons | To select the uncombined shot/repetition/average number for display |
|  | *Experiment* dropdown menu | To select the result name for display and outputs |
|  | *Result* dropdown menu | To choose the type of output results: concentration table, diffusion fitting |
|  | *Metabolite* dropdown menu | To select the metabolite name for diffusion fitting |
|  | *Update fitting* button | To update diffusion fitting after changing max b-values |
|  | *Diffusion fitting* graph | To show stick/kurtosis diffusion fitting |
|  | *Data index* box | To show the order of input datasets and rearrange if needed |
|  | *3D plot* checkbox | To switch the spectral display to 3D mode |
|  | *Scale Min* & *Max* boxes | To adjust the y-axis scale if needed |
|  | *Type* dropdown menu | To select the type of data/results for display and outputs |
|  | *abs* button | To display the absolute amplitude of signals |
|  | *real* button | To display the real part of the signals |
|  | *imag* button | To display the imaginary part of signals |
|  | *Data* button | To display the DWS experimental spectrum |
|  | *Fit* button | To display the LCModel fitting |
|  | *Baseline* button | To display the LCModel baseline |

**Supplementary Table 2**. List of basis sets provided with the toolbox (see details in Basis set section). All BASIS files are located in the *Demo_Data* folder with their respective methodologies and field strengths

| **Methodology** | **Field** | **Sequence Type** | **Name of Basis Set (*.BASIS)** | **Additional Information** |
| --- | --- | --- | --- | --- |
| ^1^H-MRSI | 9.4 T | FID (AD = 0 ms) | 9p4T_FID_MRSI_AD0us_Demo_Fillgaps | To use with FID dataset, with *Fillgaps* option |
| ^1^H-MRSI | 9.4 T | FID (AD = 1.3 ms) | 9p4T_FID_MRSI_AD1300us_Demo | To use with FID dataset |
| Indirect ^2^H-MRSI | 9.4 T | FID (AD = 1.3 ms) | 9p4T_FID_2H_MRSI_AD1300us_Demo | To use with Deuterium dataset |
| ^1^H-MRSI | 9.4 T | SE (TE = 3.3 ms) | 9p4T_SE_MRSI_TE3ms_Demo | To use with SE dataset |
| ^1^H-MRSI | 14.1 T | FID (AD = 1.3 ms) | 14p1T_FID_MRSI_AD1300us_Demo | To use with FID dataset |
| ^1^H-SVS | 9.4 T | STEAM (TE = 3 ms) | 9p4T_STEAM_SVS_TE3ms_Demo | To use with STEAM dataset |
| ^1^H-SVS | 14.1 T | SPECIAL (TE = 9.3 ms) | 14p1T_SPECIAL_SVS_TE9ms_Demo | To use with SPECIAL dataset |
| ^1^H-DWS | 9.4 T | dSPECIAL (TE = 9.5 ms) | 9p4T_dSPECIAL_DWS_TM150ms_Demo | To use with dSPECIAL dataset |
| ^1^H-DWS | 14.1 T | dSPECIAL (TE = 9.5 ms) | 14p1T_dSPECIAL_DWS_TM40ms_Demo | To use with dSPECIAL dataset |

**Supplementary Table 3**. Representative MRSI output: Hippocampus’s concentration table of 9.4 T FID-MRSI data processed with no additional processing

| **Metabolite** | **Mean relative concentration (a.u.)** | **Standard deviation (a.u.)** | **No. voxels** |
| --- | --- | --- | --- |
| Mac | 0.0006 | 0.0001 | 46 |
| Cr | 3.7802 | 0.3605 | 37 |
| PCr | 4.3599 | 0.4819 | 42 |
| Ins | 8.0133 | 0.7241 | 45 |
| NAA | 10.2189 | 0.8034 | 46 |
| Tau | 5.8674 | 1.4941 | 46 |
| PCho | 1.7725 | 0.3297 | 8 |
| GPC | 1.5543 | 0.3268 | 3 |
| Glu | 9.7928 | 1.5043 | 46 |
| Gln | 2.9545 | 0.9671 | 39 |
| Ala | 2.5590 | 0.0000 | 1 |
| Asc | 4.2571 | 0.6209 | 38 |
| Asp | 4.9795 | 0.8754 | 36 |
| GABA | 2.6505 | 0.5938 | 25 |
| Glc | 5.7495 | 1.2408 | 28 |
| GSH | 1.9452 | 0.3363 | 36 |
| Lac | 2.6736 | 0.6741 | 35 |
| NAAG | 2.1719 | 1.0195 | 42 |
| PE | 0.0000 | 0.0000 | 0 |
| NAA+NAAG | 12.3467 | 1.5545 | 46 |
| Glu+Gln | 12.6337 | 2.2019 | 46 |
| GPC+PCho | 2.0451 | 0.3414 | 46 |
| Cr+PCr | 8.0000 | 0.0000 | 46 |

**Supplementary Table 4**. Representative SVS output: Concentration table of 9.4 T STEAM SVS data processed with spectral alignment and outlier removal

| **Metabolite** | **Relative concentration (a.u.)** | **“Absolute” concentration (mmol/kg)** | **CRLB (%)** |
| --- | --- | --- | --- |
| Mac | 0.000 | 0.000 | 2 |
| Cr | 4.025 | 4.362 | 4 |
| PCr | 3.975 | 4.308 | 4 |
| NAA | 8.937 | 9.686 | 2 |
| Tau | 6.697 | 7.258 | 3 |
| PCho | 0.635 | 0.688 | 24 |
| GPC | 0.405 | 0.439 | 36 |
| Glu | 9.011 | 9.767 | 3 |
| Gln | 3.204 | 3.473 | 6 |
| Ala | 0.623 | 0.675 | 20 |
| Asc | 2.622 | 2.842 | 9 |
| Asp | 1.642 | 1.780 | 19 |
| GABA | 1.079 | 1.170 | 14 |
| GSH | 0.846 | 0.917 | 13 |
| Lac | 2.035 | 2.206 | 7 |
| Ins | 6.839 | 7.412 | 3 |
| NAAG | 0.898 | 0.973 | 10 |
| PE | 1.623 | 1.759 | 13 |
| BHB | 0.120 | 0.130 | 92 |
| NAA+NAAG | 9.835 | 10.659 | 2 |
| Glu+Gln | 12.216 | 13.240 | 3 |
| GPC+PCho | 1.040 | 1.127 | 5 |
| Cr+PCr | 8.000 | 8.671 | 2 |

**Supplementary Table 5**. Representative DWS output: “Concentration” table of 9.4 T dSPECIAL DWS data (b-value 1) processed with ISIS handling, spectral alignment, and outlier removal

| **Metabolite** | **Relative concentration (a.u.)** | **“Absolute” concentration (a.u.)*** | **CRLB (%)** |
| --- | --- | --- | --- |
| Mac | 0.0015 | 0.0000 | 3 |
| Cr | 3.9010 | 0.0136 | 5 |
| PCr | 4.0990 | 0.0143 | 4 |
| Ins | 6.3830 | 0.0222 | 3 |
| NAA | 9.4710 | 0.0330 | 1 |
| Tau | 4.3300 | 0.0151 | 3 |
| PCho | 1.0310 | 0.0036 | 5 |
| GPC | 0.0000 | 0.0000 | 999 |
| Glu | 9.1380 | 0.0318 | 2 |
| Gln | 3.0320 | 0.0106 | 5 |
| Ala | 0.0850 | 0.0003 | 120 |
| Asc | 2.5410 | 0.0088 | 8 |
| Asp | 1.4730 | 0.0051 | 24 |
| GABA | 1.2110 | 0.0042 | 13 |
| Glc | 2.3680 | 0.0082 | 12 |
| GSH | 0.8150 | 0.0028 | 12 |
| Lac | 1.1020 | 0.0038 | 9 |
| NAAG | 0.3220 | 0.0011 | 29 |
| PE | 2.1230 | 0.0074 | 11 |
| NAA+NAAG | 9.7930 | 0.0341 | 1 |
| Glu+Gln | 12.1690 | 0.0423 | 2 |
| GPC+PCho | 1.0310 | 0.0036 | 5 |
| Cr+PCr | 8.0000 | 0.0278 | 1 |

* No reference water data is specified; therefore, “absolute” “concentration” values are in arbitrary units.
